# A Polymorphism of Bactericidal/Permeability-Increasing Protein Affects Its Neutralization Efficiency towards Lipopolysaccharide

**DOI:** 10.3390/ijms23031324

**Published:** 2022-01-25

**Authors:** Katharina U. Ederer, Jonas M. Holzinger, Katharina T. Maier, Lisa Zeller, Maren Werner, Martina Toelge, André Gessner, Sigrid Bülow

**Affiliations:** Institute of Clinical Microbiology and Hygiene, University Hospital Regensburg, Franz-Josef-Strauß-Allee 11, 93053 Regensburg, Germany; katharina.ederer@ukr.de (K.U.E.); jonas.holzinger@ukr.de (J.M.H.); katharina1.maier@ukr.de (K.T.M.); lisa.zeller@ukr.de (L.Z.); maren.werner@ukr.de (M.W.); martina.toelge@ukr.de (M.T.); andre.gessner@ukr.de (A.G.)

**Keywords:** bactericidal/permeability-increasing, lipopolysaccharide, sepsis, single nucleotide polymorphism

## Abstract

Gram-negative sepsis driven by lipopolysaccharide (LPS) has detrimental outcomes, especially in neonates. The neutrophil-derived bactericidal/permeability-increasing protein (BPI) potently neutralizes LPS. Interestingly, polymorphism of the *BPI* gene at position 645 (rs4358188) corresponds to a favorable survival rate of these patients in the presence of at least one allele 645 A as opposed to 645 G. When we exploited the existing X-ray crystal structure, the corresponding amino acid at position 216 was revealed as surface exposed and proximal to the lipid-binding pocket in the N-terminal domain of BPI. Our further analysis predicted a shift in surface electrostatics by a positively charged lysine (BPI_216K_) exchanging a negatively charged glutamic acid (BPI_216E_). To investigate differences in interaction with LPS, we expressed both BPI variants recombinantly. The amino acid exchange neither affected affinity towards LPS nor altered bactericidal activity. However, when stimulating human peripheral blood mononuclear cells, BPI_216K_ exhibited a superior LPS-neutralizing capacity (IC_50_ 12.0 ± 2.5 pM) as compared to BPI_216E_ (IC_50_ 152.9 ± 113.4 pM, *p* = 0.0081) in respect to IL-6 secretion. In conclusion, we provide a functional correlate to a favorable outcome of sepsis in the presence of BPI_216K_.

## 1. Introduction

Sepsis is a life-threatening disorder with a high global burden. In 2017, approximately 19.7% of recorded global deaths were related to sepsis [1]. Since almost half of the reported cases occurred in children younger than five years, pediatric sepsis is of special concern [1]. In particular, the outcome of neonatal sepsis is detrimental with an estimated 17.6% of patients dying [2]. Sepsis is characterized by a dysregulated and overwhelming host immune response towards invading pathogens and their pathogen-associated molecular patterns (PAMPs). In Gram-negative infection, lipopolysaccharide (LPS) is a highly immunostimulatory PAMP known for driving sepsis [3,4].

Bactericidal/permeability-increasing protein (BPI) is a neutrophil-derived cationic protein with bactericidal activity towards Gram-negative bacteria [5,6,7] and is known for its potent anti-inflammatory, LPS-neutralizing activity [8]. Neutralization of LPS has been attributed to the highly cationic N-terminal tip of BPI [9]. However, a contribution of two lipid-binding pockets found in both the N-terminal and C-terminal barrel of the protein has been considered [10,11]. Potent inhibition of LPS-induced TNF secretion by BPI has been shown in vitro when added to human whole blood [12]. Moreover, BPI also attenuated the LPS-induced release of pro-inflammatory interleukin (IL)-6 and TNF in vivo in rats and humans [13,14]. Albeit the death rate was high even before supplementation could be started, the administration of an N-terminal fragment of BPI significantly decreased the amputation rate and tended to decrease the mortality of children with severe meningococcal sepsis [15]. Importantly, clinical studies have indicated an impact of the single nucleotide polymorphism (SNP) rs4358188 within the *BPI* gene in the context of sepsis. Thereby, an exchange of guanine (G) to adenine (A) at nucleotide 645 leads to the substitution of glutamic acid (BPI_216E_) with lysine (BPI_216K_) at amino acid position 216. Premature neonates heterozygous for both BPI variants had a significantly lower risk for developing sepsis compared to those homozygous for BPI_216E_ [16]. Congruently, in an independent cohort of neonatal sepsis, nine out of 14 non-survivors (64.3%) were homozygous for BPI_216E_ opposed to 76 of the 315 surviving patients (24.1%), also indicating a higher frequency of BPI_216E_ in sepsis with lethal outcomes (calculated according to data published in [17]).

This study, exploiting an existing X-ray crystal structure of BPI, revealed that amino acid 216 is proximal to the lipophilic binding pocket at the N-terminal domain implicating a potential effect on the binding to the negatively charged core of LPS. Thus, we compared the functional properties of the two BPI variants with special emphasis on bactericidal, LPS-binding, and LPS-neutralizing properties. We provide new evidence that an exchange of amino acids with an opposing charge proximal to the lipid-binding pocket enhances LPS neutralization by BPI, thus, explaining favorable outcomes in sepsis. This finding has an impact on the risk stratification of sepsis patients and supports the substitution of BPI especially in patients homozygous for BPI_216E_.

## 2. Results

### 2.1. Models of BPI_216E_ and BPI_216K_ Reveal Different Charge Distributions Proximal to the Lipid Binding Pocket

**Figure 1:**
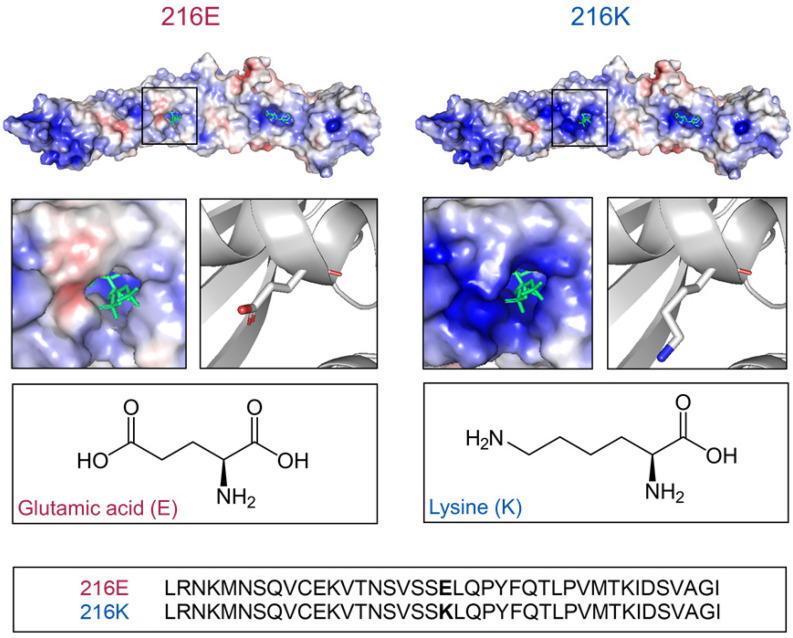
Comparison of the surface charge surrounding the N-terminal apolar lipid-binding pockets of BPI_216E_ and BPI_216K_. Three-dimensional modeling and electrostatic potential of BPI_216E_ and BPI_216K_ with red areas representing negative and blue areas representing positive charges. Bound phosphatidylcholine is shown in green. Detailed view of the N-terminal binding pocket and zoomed-in ribbon diagram indicating the glutamic acid and lysine residues of BPI_216E_ and BPI_216K_, respectively. Amino acid sequences for BPI_216E_ and BPI_216K_ from position 196 to 236 are shown.

The interaction of BPI with the acyl chains of LPS has been proposed to be mediated by the apolar binding pockets found in the C-terminal and N-terminal domains of the protein [10,11]. Interestingly, the SNP rs4358188 localizes proximal to the entrance of the N-terminal lipid-binding pocket where it results in a substantial electrostatic change. While BPI_216E_ with a glutamic acid at position 216 displays a partially negative charge, rs4358188 determines an exchange to lysine in BPI_216K_ with a consecutive positive charge (Figure 1). Thus, the SNP at codon position 216 leads to a BPI variant with a distinct change in the charge of surface patches surrounding the N-terminal apolar binding pocket.

### 2.2. BPI_216E_ and BPI_216K_ Do Not Differ in Bactericidal Activity

Both a 25 kDa N-terminal and a 30 kDa C-terminal fragment of BPI are capable of LPS neutralization, whereas only the N-terminal fragment was described to harbor antibacterial activity [12]. To analyze if the differences in electrostatics at the N-terminal lipid-binding pocket of the BPI variants influence the bactericidal and LPS neutralizing activities of BPI, we performed bacterial killing and LPS binding assays.

**Figure 2:**
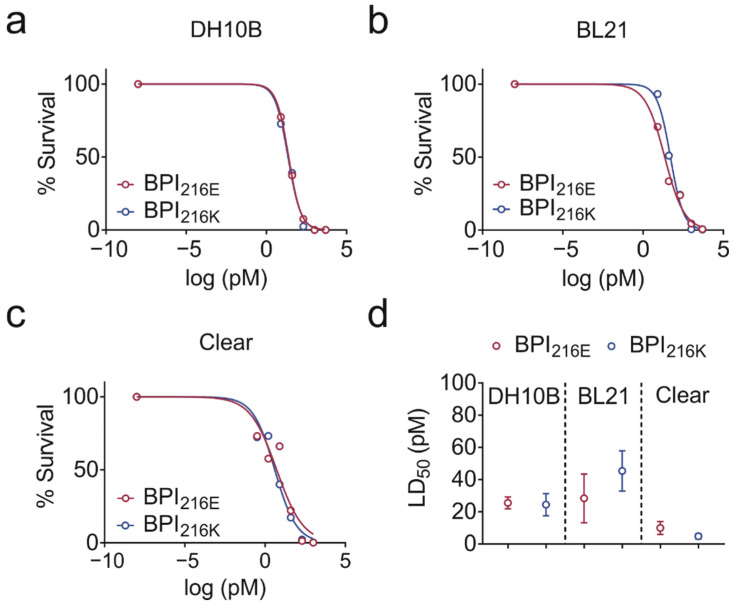
Bactericidal activity of BPI_216E_ and BPI_216K_ towards *E. coli*. (**a**–**c**) Dose-response experiments with *E. coli* strains DH10B (**a**), BL21 (**b**), and Clear Coli^®^ BL21 (**c**) incubated with increasing concentrations of BPI_216E_ and BPI_216K_. (**d**) LD_50_ is depicted for each tested *E. coli* strain. Colony numbers of untreated bacteria were set as a reference to 100% (**a**–**c**). Data are shown as the means (**a**–**c**) or means ± SEM (**d**) of three biological replicates. Student’s ratio paired *t*-test revealed no significant differences.

BPI_216E_ and BPI_216K_ were equally potent at inhibiting the growth of *E. coli* DH10B and BL21 in a concentration-dependent manner (Figure 2a,b) as indicated by the mean lethal dose (LD_50_) of BPI_216E_ and BPI_216K_ towards *E. coli* DH10B (25.6 pM ± 3.7 and 24.4 pM ± 6.9) and *E. coli* BL21 (28.4 pM ± 15.1 and 45.4 pM ± 12.5; Figure 2d). We also tested the LD_50_ of the two variants towards Clear Coli^®^ BL21, which expresses a genetically modified LPS variant consisting of lipid IVA, i.e., a lipid A precursor lacking the 2′ and 3′ acyl chain and associated oligosaccharides [18]. In this context, previous data demonstrated that BPI binding to the lipid A portion of LPS depends on the number of lipid A acyl chains and that long oligosaccharide chains of membrane-anchored LPS sterically hinder BPI from binding to the bacterial membrane [19,20,21]. In accordance, the LD_50_ was lower towards Clear Coli^®^ BL21 than towards strains DH10B and BL21 but independent of the BPI variant with an LD_50_ of 10.0 pM ± 4.0 for BPI_216E_ and 4.8 pM ± 1.7 for BPI_216K_ (Figure 2c,d). Summarizing, no difference in bactericidal activity towards different *E. coli* strains was observed for the BPI variants.

### 2.3. BPI_216K_ Does Not Exhibit Higher Affinity to LPS as Compared to BPI_216E_

**Figure 3:**
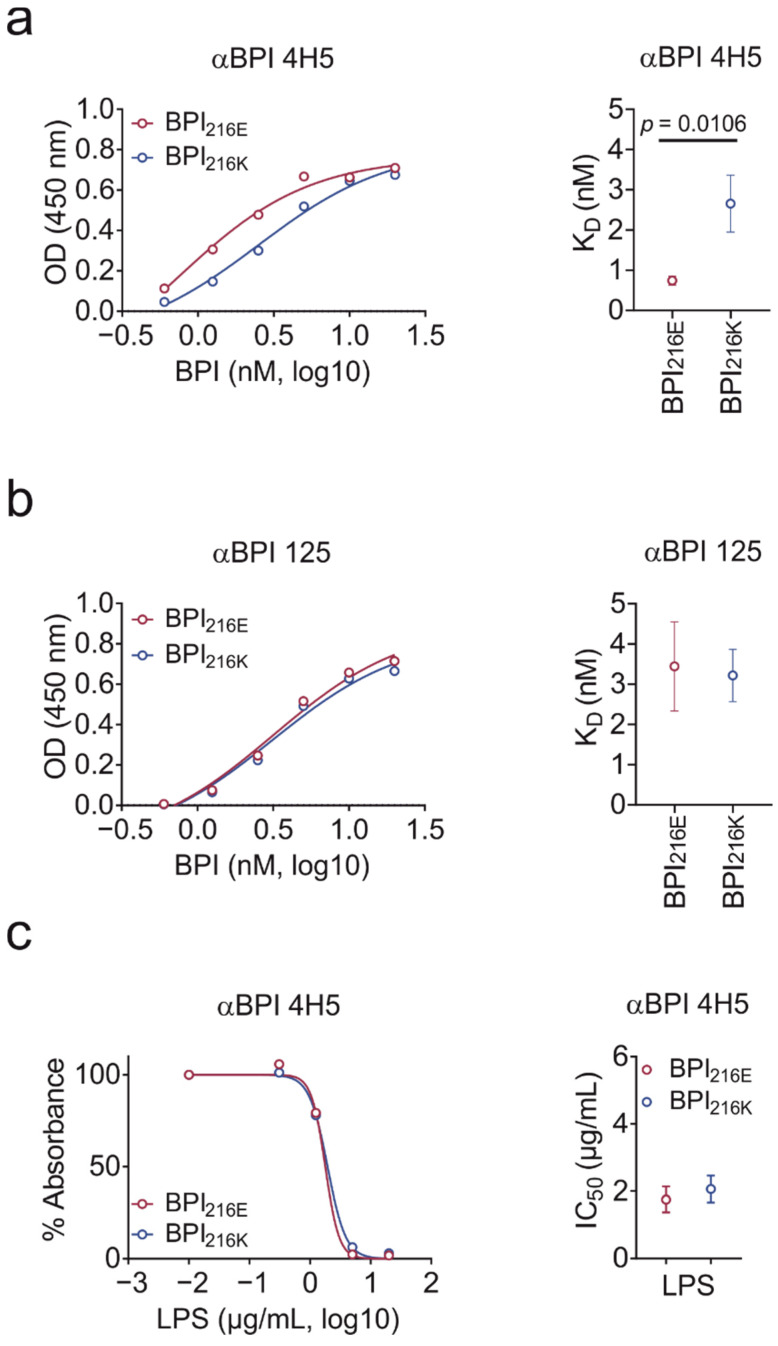
Equal binding of BPI variants to LPS. (**a**,**b**) Binding of BPI to solid-phase LPS as detected with αBPI antibody clone 4H5 (**a**) or αBPI antibody 125 newly generated in mice (**b**). K_D_ values are provided. (**c**) BPI_216E_ and BPI_216K_ were pre-incubated with increasing concentrations of liquid-phase LPS before adding the mixture to LPS-coated plates. IC_50_ values are depicted. Absorbance measured for binding of BPI to the LPS-coated plate without pre-incubation with liquid-phase LPS was set as a reference to 100% (**c**). Data are shown as the means (**left**) or means ± SEM (**right**) of three biological replicates. Statistical testing was performed using the Student’s ratio paired *t*-test. Significance is indicated by the *p* value.

Next, we compared the binding capacity of BPI_216E_ and BPI_216K_ towards solid-phase LPS. LPS binding affinity was significantly different for BPI_216E_ and BPI_216K_ when detecting BPI binding with αBPI clone 4H5 (Figure 3a). Unexpectedly, the calculated K_D_ of BPI_216K_ towards negatively charged LPS was higher than for BPI_216E_ despite disadvantageous charge distribution. The binding site of clone 4H5 to BPI is not known. However, the sequence and/or possible consecutive conformational differences between BPI_216E_ and BPI_216K_ could result in different binding affinities of antibody 4H5 towards the variants. Therefore, we repeated the test with a second, newly generated αBPI antibody (αBPI clone 125, Figure 3b). Importantly, the difference in BPI_216E_ and BPI_216K_ towards LPS binding disappeared. To retest clone 4H5, we performed an inhibition assay by pre-incubating BPI with liquid-phase LPS and set absorbance measured for binding of BPI to the LPS-coated plate without pre-incubation to 100%. In accordance with the results for clone 125, liquid-phase LPS inhibited binding of BPI_216E_ and BPI_216K_ to solid-phase LPS in a comparable extent (Figure 3c). Summarizing, despite having opposing charges proximal to the lipid-binding pocket, BPI_216K_ does not exhibit superior binding towards the negatively charged LPS as compared to BPI_216E_.

### 2.4. BPI_216K_ Exceeds BPI_216E_ at Inhibition of LPS-Induced Cytokine Secretion

**Figure 4:**
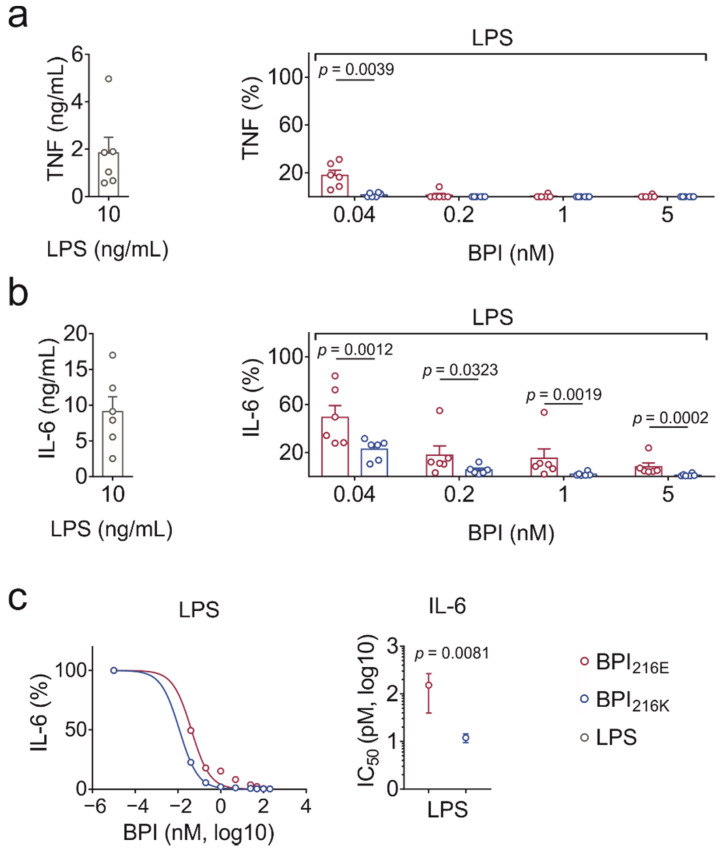
Comparison of the LPS neutralizing capacity of BPI_216E_ and BPI_216K_. (**a**,**b**) Levels of TNF and IL-6 in supernatants of human PBMCs after 24 h of stimulation with LPS (10 ng/mL) ± BPI. (**c**) Dose–response curve and IC_50_ of BPI_216E_ and BPI_216K_ as shown for IL-6 secretion. Cytokine secretion for LPS alone was set as a reference to 100% (**a**,**b**, right panel, as well as **c**, left panel). Data are shown as the means (**c**, left panel) or means ± SEM (**a**,**b**, both panels, and **c**, right panel) of six biological replicates. Statistical testing was performed using the Student’s ratio paired *t*-test. Significance is indicated by *p* values.

To evaluate the LPS neutralization capacity, human peripheral blood mononuclear cells (PBMCs) were incubated with either BPI variant and increasing concentrations of LPS for 24 h before cytokine response was determined in the cell culture supernatants. Both BPI variants were more potent in neutralizing TNF than IL-6 (Figure 4a,b). For both cytokines, BPI_216E_ was significantly less potent than BPI_216K_ in LPS neutralization (Figure 4a,b). Since the results became too variable at BPI concentrations lower than those indicated, the IC_50_ could only be determined for IL-6. Hereby, extrapolation revealed an IC_50_ of 152.9 ± 113.4 pM for BPI_216E_ and significantly lower values of 12.0 ± 2.5 pM for BPI_216K_ (Figure 4c). Therefore, on average, BPI_216K_ exhibited a more than ten-fold higher capacity to neutralize LPS in PBMC culture than BPI_216E_.

## 3. Discussion

The reduced LPS-neutralization capacities of BPI_216E_ compared to BPI_216K_ shown in this study provide an explanation for an increased susceptibility to Gram-negative sepsis and mortality in neonates in association with BPI_216E_ [16,17]. Because of the differently charged amino acids located proximal to the lipid-binding site in the N-terminal domain of BPI, we initially expected differences in binding of BPI_216E_ and BPI_216K_ to LPS. However, no conclusive difference in respect to the binding of LPS by either BPI variant was detected, indicating a minor contribution of amino acid 216. Moreover, both variants displayed equal bactericidal activity independent of oligosaccharide chains or the number of acyl chains in lipid A of the tested strains. Since BPI_216E_ neutralizes LPS less efficiently than BPI_216K_, BPI_216E_ presumably enables a more sensitive perception of bacterial invasion. In this context, the SNP at position 216 K is associated with a predisposition to bacterial infection in hematopoietic stem cell transplant (HSCT) patients [22]. Additionally, BPI_216E_ was linked to an increased risk of graft versus host disease (GvHD) after HSCT [23], possibly attributed to the key role of LPS in the pathophysiology of GvHD [24,25].

Although the elevation of sepsis frequency and association with increased mortality was seen in pediatric patients with variant BPI_216E_ [16,17], no difference was found in an adult cohort study [26]. Neonatal neutrophilic granulocytes showed reduced levels [27,28] and extrusion [29] of BPI in neutrophilic granulocytes compared to adults. Lower basal levels of BPI may lead to a more pronounced phenotype of the respective BPI variants, possibly explaining divergent effects seen for children and adults. However, low patient numbers may limit the validity of the studies in neonates, and an analysis of larger cohorts is needed. LPS is recognized by toll-like receptor (TLR) 4 [30]. The combination of rs4358188 with SNPs in genes related to TLR-related pathways, namely *IL1RL* and *ITGB2*, seems to significantly increase the risk for developing specific IgE directed against a food allergen [31], thereby attributing a higher risk in the presence of BPI_216K_. Fittingly, LPS can ameliorate ongoing allergic inflammation in dependence of TLR4 in murine models [32].

A previous study performed in whole blood stimulated with LPS at 1 ng/mL showed an IC_50_ for TNF at a BPI concentration of 4 nM [12]. Our use of PBMCs in serum-free conditions revealed LPS neutralization by BPI even at a picomolar range, emphasizing the enormous potential of BPI to neutralize LPS. A limitation of our study is that we did not provide a mechanistic insight concerning the difference in neutralization potency between the variants. This must be addressed in further studies. Although K_D_ towards LPS was comparable, distinct association and dissociation rates might possibly explain our findings. The charge of amino acid 216 might also contribute to conformational changes in BPI which were previously found for the N-terminal part of BPI upon binding to LPS membranes [33]. In addition, the described maximum binding capacity of 40 LPS molecules per BPI might be affected [34]. Since BPI polymorphism possibly influences a variety of diseases including sepsis, GvHD, and allergy, in vivo models would be of interest. Compared to humans, constitutive BPI expression in mice is impaired [35] and murine BPI was shown to exhibit lower bactericidal and LPS-neutralization capacities than human BPI [36]. However, BPI-deficient mice were recently generated [37,38] and should be suited as basic tools for the comparison of therapeutically applied BPI_216E_ and BPI_216K_ in different disease conditions.

In conclusion, the decreased LPS-neutralizing capacity of BPI_216E_ as compared to BPI_216K_ provides an explanation for the association of BPI_216E_ with the risk of unfavorable outcomes in sepsis [16,17]. Lethality in this patient group during the course of Gram-negative sepsis is high [2] and the application of a recombinant N-terminal domain of BPI showed partial clinical success in children with meningococcal infection [14,15]. Given the rise in multidrug-resistant Gram-negative bacteria, the administration of BPI during Gram-negative sepsis, especially in patients with a BPI_216E_ phenotype and/or BPI deficiency of various causes [39], should be re-evaluated in clinical studies.

## 4. Materials and Methods

### 4.1. Generation of Recombinant Human BPI

The generation of recombinant human BPI variants was performed as described [40] with slight modifications. In brief, a pCR3 vector (Invivogen, Toulouse, France) construct comprising an N-terminal HA signal peptide, amino acids 32 to 487 of either BPI_216E_ or BPI_216K_, and a C-terminal FLAG-Tag was transfected in Expi293F™ cells using the ExpiFectamine™ 293 Transfection Kit (Thermo Fisher Scientific, Waltham, MA, USA). The expressed protein was purified by cation exchange chromatography via a HiTrap™ SP HP column (Cytiva, Marlborough, MA, USA). Fractions containing the protein of interest were pooled and purified by size exclusion chromatography using a HighLoad 16/600 Superdex 75 pg column (Cytiva, Marlborough, MA, USA), concentrated via ultrafiltration (Amicon Ultra-15, Merck Millipore, Darmstadt, Germany), and dialyzed against PBS. Concentration was determined by DC-Protein Assays (Bio-Rad Laboratories, Feldkirchen, Germany).

### 4.2. Generation and Purification of BPI Antibodies

Mice were immunized with recombinant BPI (amino acids 32 to 487) by Davids Biotechnology (Regensburg, Germany) to generate hybridoma clones. These clones were screened by ELISA for the production of antibodies directed against human BPI. The positive IgG clone 125 was selected for expansion and the antibody was purified via a HiTrap Protein G HP antibody purification column (Cytiva, Marlborough, MA, USA).

### 4.3. Solid-Phase BPI Binding Assay

The setup for the BPI binding assay was adopted from Bülow et al., 2018 [40]. To summarize, streptavidin-coated 96-well plates (Nunc^TM^ Immobilizer^TM^ Streptavidin F96 clear, Thermo Fisher Scientific, Waltham, MA, USA) were coated with biotinylated LPS derived from *E. coli* O111:B4 (2 μg/mL; Invivogen, Toulouse, France) in PBS and agitated overnight at 23 °C and 350 rpm. Plates were then washed with NaCl HEPES buffer (150 mM NaCl and 50 mM HEPES) containing 0.01% casein (Applied Biosystems, Waltham, MA, USA). After blocking with 1% BSA at 37 °C and three washing steps, plates were incubated with BPI at concentrations as indicated. For inhibition experiments, 20 nM BPI was pre-incubated with *E. coli* O111:B4-derived LPS (Invivogen, Toulouse, France) in NaCl HEPES buffer containing 0.1% BSA for 30 min using LoBind tubes (Eppendorf, Hamburg, Germany). After washing, bound BPI was detected by murine anti-human BPI monoclonal antibody 4H5 (Hycult Biotech, Uden, The Netherlands) or antibody 125 followed by detection via an HRP-conjugated rabbit anti-mouse IgG (Dianova, Hamburg, Germany). TMB (BD Biosciences, Heidelberg, Germany) was used as a substrate of the peroxidase. The reaction was stopped after seven minutes with 1 N HCl (Carl ROTH, Karlsruhe, Germany). Absorbance was then measured at 450 nm in a microplate reader (Model 550, Bio-Rad Laboratories, Feldkirchen, Germany).

### 4.4. Dose Response Experiments for Bactericidal Activity

*E. coli* strains DH10B (Invitrogen, Carlsbad, CA, USA), BL21 (Lucigen, Middleton, WI, USA), and Clear Coli^®^ BL21 (Lucigen, Middleton, WI, USA) were cultivated on Columbia blood agar plates (Thermo Fisher Scientific, Waltham, MA, USA). Single colonies were transferred into lysogeny broth medium (Carl Roth, Karlsruhe, Germany) and incubated overnight at 37 °C at 220 rpm. Subsequently, the optical density (OD) of the bacteria was adjusted to 0.1. Inoculated broths were further incubated until an OD of 0.4 was reached. Bacteria were then pelleted and diluted in PBS with 0.01% Tween 80 (Merck Millipore, Darmstadt, Germany) to a final concentration of 1 × 10^4^ bacteria per mL. Bacterial suspensions were then incubated with decreasing concentrations of BPI for 1 h at 37 °C and an aliquot, containing 5 × 10^2^ bacteria, was immediately plated on blood agar plates. Plates were incubated overnight at 37 °C and colonies were quantified the next day.

### 4.5. Isolation of Human Peripheral Blood Mononuclear Cells

For the isolation of PMBCs, blood was drawn from healthy male volunteers and collected in heparinized blood collectors (Li-Heparin-Gel-Monovette, Sarstedt, Nümbrecht, Germany). Blood was then diluted in RPMI-1640 (Sigma-Aldrich, Taufkirchen, Germany) and centrifuged in Leucosep^TM^ tubes containing FICOLL^®^ PAQUE PLUS (Oxford Immunotec, Abingdon, UK) at 1000× *g* for 10 min. PBMCs were then isolated from the interphase and washed two times. Subsequently, the pellet was resuspended in AIM V^®^ Medium (Thermo Fisher Scientific, Waltham, MA, USA) and 5 × 10^4^ cells were seeded into a 96-well plate at a final volume of 100 µL. PBMCs were allowed to rest for 4 h prior to combined stimulation with BPI and LPS.

### 4.6. Quantification of Cytokine Levels

To quantify cytokine levels in the supernatants of stimulated PBMCs, the Luminex^®^ 100 system was used (Austin, TX, USA). Antibodies to detect human IL-6 (human IL-6 ELISA set) and TNF (human TNF ELISA set) were purchased from BD Bioscience (Heidelberg, Germany). Measured concentrations were calculated by using LiquiChip Analyzer Software (Qiagen, Hilden, Germany).

### 4.7. Structure Modeling, Graphical Depictions, and Statistics

The PDB structure for human BPI_216E_ (10.2210/pdb1EWF/pdb [41]) was mutated in PyMOL (PyMOL Molecular Graphics System, Version 2.3.2 Schrödinger, LLC., New York, NY, USA) to BPI_216K_. Rendering of three-dimensional structures was performed in PyMOL. Electrostatic surface potentials for BPI_216E_ and BPI_216K_ were calculated with the APBS plugin [42] for PyMOL. Graphical depictions and statistical analyses were performed using GraphPad Prism, version 7 for Windows (GraphPad Software, San Diego, CA, USA). For comparison of BPI_216E_ and BPI_216K_, using a ratio paired *t*-test, okines values beyond the linear range of the standard curve werication of the test. To enablection of LD_50_ and IC_50_ curves on a logarithmic scale, concentrations with values of zero were represented as values at least 30-fold beyond the lowest applied concentration of the respective substance.

## Data Availability

Data are contained within the article.
